# Single Blind Randomized Controlled Trial of Modified Constraint-Induced Movement Therapy in Infants With the Sequelas of Unilateral Brachial Plexus Injury

**DOI:** 10.3389/fnhum.2022.900214

**Published:** 2022-05-30

**Authors:** Zhenzhen Cui, Le Liu, Xi Chen, Haiyan Zeng, Shizhu Zheng, De Wu

**Affiliations:** ^1^Department of Pediatrics, The First Affiliated Hospital of Anhui Medical University, Hefei, China; ^2^Department of Pediatric, Anhui Province Maternity and Child Health Hospital, Hefei, China; ^3^Pediatric Neurorehabilitation Center, Hefei Changxing Rehabilitation Hospital, Hefei, China; ^4^Pediatric Neurorehabilitation Center, Lu’an Rehabilitation Hospital, Lu’an, China

**Keywords:** modified constraint-induced movement therapy, unilateral brachial plexus injury, rehabilitation, therapy, sequelas

## Abstract

**Objective:**

To explore the effect of modified constraint-induced movement therapy (mCIMT) on upper limbs residual dysfunction for infancy with the sequelas of unilateral brachial plexus injury (uBPI).

**Methods:**

Single blind randomized controlled trial of mCIMT vs. standard care. An enrolling 31 infants with a uBPI exhibiting residual dysfunction of the affected upper limb for over 6 months was conducted. And functional outcomes pertaining to the affected upper limb were assessed *via* AMS, GRES, RHS, and MSS at 0, 3, and 6 months after treatment.

**Results:**

No differences were found in baseline (acquisition phase) AMS, MSS, GRES, or RHS between the control and mCIMT groups [*F*(1, 14) = 0.062, *P* = 0.086; *F*(1, 14) = 0.483, *P* = 0.499; *F*(1, 14) = 0.272, *P* = 0.610; *Z* = −0.336, *P* = 7.373]. At the 3- and 6-month follow-up time points, AMS, MSS, and GRES scores were significantly improved over baseline in both groups [mCIMT: *F*(2, 30) = 183.750, 128.614, 110.085, *P* < 0.05; Control: *F*(2, 28) = 204.007, 75.246, 51.070, *P* < 0.05]. No significant differences were found between two treatment groups at the 3-month follow-up time point [*F*(1, 14) = 0.565, *P* = 0.465; *F*(1, 14) = 0.228, *P* = 0.641; *F*(1, 14) = 0.713, *P* = 0.413; *Z* = −0.666, *P* = 0.505]. However, at the 6-month follow-up time point, AMS and MSS scores were significantly improved in the mCIMT group relative to the control group [*F*(1, 14) = 8.077, *P* = 0.013; *F*(1, 14) = 18.692, *P* = 0.001].

**Conclusion:**

mCIMT may benefit the rehabilitation of residual upper limb dysfunction associated with a uBPI in infants.

**Clinical Trial Registration:**

[www.chictr.org.cn], identifier [ChiCTR1900022119].

## Introduction

Brachial plexus birth injuries (BPI) occur at a frequency of 1–2 per 1,000 births (Brucker et al., 1991; Smith and Patel, 2016). Continuous external compression or traction of the head, neck, and shoulders can readily cause brachial plexus nerve injuries, which most commonly develop as a form of obstetric injury or as a result of severe trauma due to falls or compression injury (Luo et al., 2021). In most cases, BPI is transitory, with complete recovery occurring in 75–95% of cases (Prigent and Romana, 2013; Polcaro et al., 2019). However, other recent studies suggest a lower total recovery rate of just 66%, with 20–30% of affected individuals experiencing residual deficits and 10–15% of cases resulting in considerably altered functionality (Hoeksma et al., 2004; Sibbel et al., 2014).

Internal rotation contractures and posterior humeral subluxation are the most prevalent long-term complications in BPI (Heise et al., 2015; Smith et al., 2018), and shoulder stiffness with internal rotation is commonly observed in the context of partial recovery with or without nerve surgery (Goubier et al., 2019). For infancy in rapid Developmental stages, injury of brachial plexus is more serious than in adults, except for long-term complications of upper limb, but also secondary lesions such as short upper limb and small hand deformity on the injured side. Therefore, Rehabilitation methods with evidence-based are needed to improve the residual upper limb function of infants with brachial plexus injury (BPI), and to reduce the deficiencies left by surgery and existing rehabilitation methods.

Modified constraint-induced movement therapy (mCIMT) was developed as an integrated treatment strategy for stroke patients that seeks to rehabilitate upper limb dysfunction in affected individuals (Rocha et al., 2021). There is further evidence that mCIMT can enhance motor and sensory functionality in the upper limbs of children affected by hemiplegia, and it is thus widely utilized for the rehabilitation of hemiplegic children (Anita et al., 2013; Islam et al., 2014). In their retrospective database analysis, Zielinski et al. (2021) observed comparable improvements in bimanual performance in children with birth-related brachial plexus injuries relative to patients with unilateral cerebral palsy.

Since 2013, we have tried to treat the sequelae of BPI with mCIMT, and found that its seems to show better short-term efficacy than the existing occupation therapy combined with physiotherapy. The primary goal of this Single blind randomized controlled trial (RCT) was to analyze the efficacy of mCIMT for infants with the sequelas of unilateral brachial plexus injury (6 months after BPI, or 4 months after operation) in comparison to conventional non-constraining bimanual treatment of equal intensity.

## Methods

### Study Design

**FIGURE 1:**
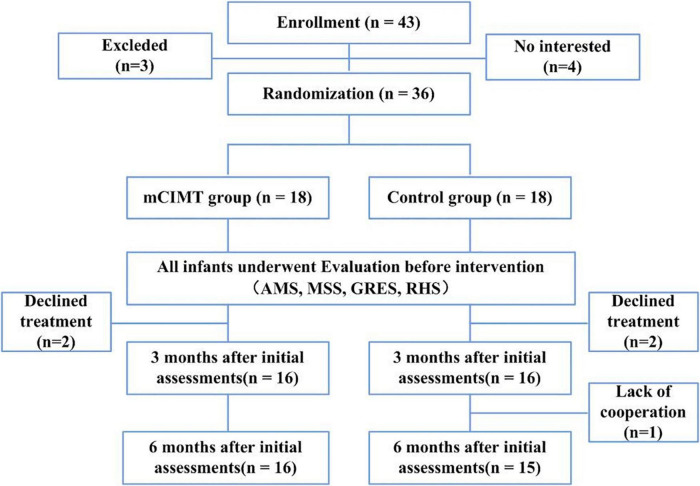
CONSORT flow diagram with number of participants and reasons for missing data in each group, at each time-point. mCIMT, modified Constraint-Induced Movement Therapy; AMS, Active Movement Scale; MSS, Mallet Shoulder Scale; GRES, Gilbert-Raimondi Elbow Scale; RHS, Raimondi Hand Scale.

This was a prospective RCT for which the parents of all patients provided written informed consent to participate. Registration number in Chinese Clinical Trial Registry (ChiCTR) is ChiCTR1900022119. In total, 43 children were considered for study participation, of whom 3 failed to meet with study inclusion criteria, while the parents of 4 children refused to participate in the study. The overall recruitment process and study flow chart are outlined in Figure 1. Children were randomly assigned to two groups using sealed envelopes prepared by a therapist that indicated whether the child was enrolled in the control or mCIMT group. Assignment was performed prior to baseline assessments by an individual who was blinded to study details and who was instructed to pick one sealed envelope containing the numbers of participating children.

### Participants

In total 36 children (14 male, 22 female) between the ages of 7–36 months with a uBPI were recruited from the Children’s Neurological Rehabilitation Center of the First Affiliated Hospital of Anhui Medical University, Hefei Changxing Rehabilitation Hospital and Lu’an Rehabilitation Hospital from July 2016 to July 2020.

Children were eligible for study inclusion if: (1) they had a confirmed diagnosis of uBPI; (2) they either (a) had electromyographic evidence of brachial plexus injury but were not eligible for surgery and exhibited upper limb motor dysfunction that had been present for over 6 months (b) exhibited clinical and electromyographic indications of neurological dysfunction more than 4 months after brachial plexus repair; and (3) their parents/guardians had provided written informed consent for study participation.

Children were excluded from this study if: (1) exhibited full-arm brachial plexus injuries requiring surgical intervention; (2) exhibited upper arm fractures or trauma; (3) presented with visual problems that would interfere with their ability to perform the intervention; or (4) diagnosed with systemic diseases with the potential to impact study outcomes, including genetic diseases, congenital deformities, or serious organ dysfunction. All parents/guardians were provided with an oral and written description of the study prior to providing written informed consent for study participation.

### Interventions

The healthy side of patients in the control group is not subject to any restrictions, and they underwent conventional rehabilitation that consisting of physical factor therapy, occupational game training, muscle strength training, and sensory stimulation.

**FIGURE 2:**
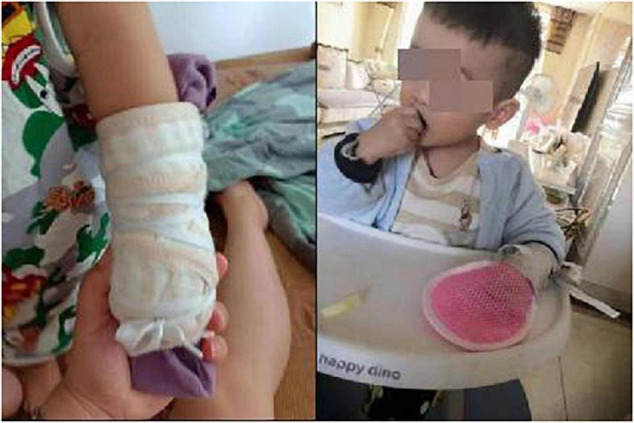
Healthy hands were restrained with suspension straps and mittens.

Patients in the mCIMT group were designed special rehabilitation plan according to the principles restrictive induction therapy (Knapp et al., 1963; Gert et al., 2015). First, suspension straps and mittens were used to restrain the healthy hands of these participants (Figure 2). Second, Two to three appropriate plastic movements were selected for 30 min at time, with a personalized rehabilitation training plan having been developed by therapists in cooperation with parents based on the specific characteristics of each individual child (The mCIMT protocol consisted of repetitive task-oriented training and adherence-enhancing behavioral strategies). Third, children were encouraged to explore and solve problems with the potential to promote motor learning (motor learning strategies were used to conduct intensive training). Exercise prescription: mCIMT, 4 h per day, 90 days; occupational game training, 0.5 h for the healthy side, 90 days (In order to eliminate the effect of long restrictive induction time on contralateral motor development).

### Outcomes

#### Active Movement Scale

The AMS is an ordinal 8-point scale (score: 0–7) that is used for the objective evaluation of the activation of extremity muscle groups as a means of measuring changes in the movement of the upper limbs. The AMS can be used to assess children of any age without the need for tasks to be performed on command. Higher scores correspond to better upper limb muscle strength (Curtis et al., 2002).

#### Mallet Shoulder Scale

The MSS is used to assess shoulder function in infants diagnosed with brachial plexus palsy, and consists of five categories: global abduction, global external rotation, hand to neck, hand to mouth, and hand to spine. Each of these categories is graded from 1 to 3, with total scores of 5 and 15, respectively, corresponding to bad and good shoulder function (Joshua and Scott, 2014).

#### Gilbert-Raimondi Elbow Scale

The GRES is employed to assess elbow functionality in infants diagnosed with a brachial plexus nerve injury. The scale assesses elbow flexion (no or some contraction = 1, incomplete flexion = 2, complete flexion = 3), elbow extension (no extension = 0, weak extension = 1, good extension = 2), and extension deficit (0–30° = 0, 30–50° = 1, More than 50° = 2). Higher scores correspond to better elbow function (Blaauw et al., 1998; Praveen and Navin, 2009).

#### Raimondi Hand Scale

The RHS scale was developed by the Brachial Plexus Workgroup, and assessed patient hand function on a 0–5 scale, with higher scores corresponding to better function. Specifically, a score of 0 is indicative of complete paralysis or slight finger flexion without utility, a lack of thumb use, an inability to pinch, and little or no sensation. A score of 1 was indicative of limited active finger flexion with no wrist or finger extension and the potential for lateral thumb pinching. A score of 2 was indicative of active wrist extension with passive finger flexion (tenodesis) and passive lateral pinching of the thumb (pronation). A score of 3 was indicative of complete active wrist and finger flexion, thumb mobility with partial abduction-opposition, intrinsic balance, no active supination, and good potential for secondary surgery. A score of 4 was indicative of complete wrist and finger flexion, active wrist extension, weak or absent finger extension, good thumb opposition with active ulnar intrinsics, and partial pronation/supination. A score of 5 was indicative of a hand with finger extension and near-complete pronation/supination (Al-Qattan, 2003).

### Statistical Analysis

All data were analyzed using SPSS (v 25.0, IL, United States). The Shapiro-Wilk test was used to assess spatiotemporal data for symmetry in order to confirm that the results were normally distributed. When normally distributed, these data were expressed as means with standard deviations at baseline and at the 3- and 6-month follow-up time points. Changes in data over time within each group (control, mCIMT) were compared *via* repeated measured ANOVA, while continuous and categorical variables were, respectively, compared between groups *via* independent samples *T*-tests and chi-squared tests. And, rank sum test was used for grade data. *P* < 0.05 was the threshold of significance.

## Results

In total, 36 children diagnosed with uBPI were enrolled in this study and randomized into the mCIMT and control groups (*n* = 18 each). Patient baseline characteristics are detailed in Table 1. Data from two children in the mCIMT group were missing immediately after intervention, while data from one and two children in the control group were missing immediately after the intervention and at follow-up, respectively. As a result, complete data were available from 31 children, of whom 16 underwent mCIMT treatment and 15 underwent conventional rehabilitation (Figure 1).

**TABLE 1:** Demographic characteristics of the randomized participants per group and statistical comparison of the demographic characteristics.

		mCIMT	Control	*df*	χ^2^ or *t*	*P*
Obstetric history	Abnormal labor	10 (62.50%)	9 (60.00%)	1	χ^2^ = 0.020	0.886
	Normal labor	6 (37.50%)	6 (40.00%)			
Affected side	Left	5 (31.25%)	6 (40.00%)	1	χ^2^ = 0.259	0.611
	Right	11 (68.75%)	9 (60.00%)			
Type of brachial palsy	Upper plexus injury	10 (62.50%)	10 (66.67%)	1	χ^2^ = 0.059	0.809
	Lower plexus injury	6 (37.50%)	5 (33.33%)			
Gender	Male	6 (37.50%)	5 (33.33%)	1	χ^2^ = 0.059	0.809
	Female	10 (62.50%)	10 (66.67%)			
Mean age months		12.06 ± 3.71	13.27 ± 4.10	29	*T* = 0.858	0.398

Twenty-nine children completed the study evaluations on time, while two did not complete the evaluations on time at the 3- and 6-month follow-up time points, but did complete these evaluations within 1 week. Rehabilitation treatment was conducted according to the formulated training plan. Patient characteristics are summarized in Table 1. There were no significant differences in patient demographic or clinical characteristics between groups at baseline (Table 1).

Mean AMS, MSS and GRES values for patients in the mCIMT and control groups at each time point (baseline, 3-month follow-up, 6-month follow-up) are summarized in Table 2. The data of AMS, MSS, and GRES scores were all normally distributed. Considering the influence of time and group, two-factor repeated ANOVA was used to judge the influence of different interventions on GMFM score over time. By Mauchly’s spherical hypothesis test, the covariance matrix of the dependent variable was equal for the interaction term group*time (*P* > 0.05). The interaction (Group*Time) effect for groups were all significant [AMS: *F*(2, 28) = 8.340, *P* = 0.001, ES = 0.373; MSS: *F*(2, 28) = 3.650, *P* = 0.039, ES = 0.207; GRES: *F*(2, 28) = 3.535, *P* = 0.043, ES = 0.202]. Therefore, the separate effects of group and time within the two research objects were tested.

**TABLE 2:** Means (standard error) of AMS, MSS, GRES measures at each time-point, and statistical comparison (Two-factor repeated measure ANOVA).

	Baseline mean ± SE (95% CI)	3-Month follow-up mean ± SE (95% CI)	6-Month follow-up mean ± SE (95% CI)
**AMS**			
mCIMT	2.53 ± 0.24 (2.03–3.04)	4.93 ± 0.32 (4.26–5.61)	6.53 ± 0.22 (6.07–7.00)
Control	2.60 ± 0.21 (2.14–3.06)	4.60 ± 0.31 (3.95–5.26)	5.53 ± 0.27 (4.95–6.12)
Group effect	*F*(1, 14) = 0.062, *P* = 0.086, ES = 0.004	*F*(1, 14) = 0.565, *P* = 0.465, ES = 0.039	*F*(1, 14) = 8.077, ***P***** = 0.013**, ES = 0.366
Time effect	mCIMT: *F*(2, 30) = 183.750, ***P***** < 0.001**, ES = 0.925 control: *F*(2, 28) = 204.007, ***P***** < 0.001**, ES = 0.936		
Time*group	*F*(2, 28) = 8.340, ***P***** = 0.001**, ES = 0.373		
**MSS**			
mCIMT	6.00 ± 0.28 (5.41–6.59)	9.73 ± 0.71 (8.22–11.25)	13.67 ± 0.53 (12.53–14.81)
Control	5.73 ± 0.25 (5.20–6.27)	9.33 ± 0.62 (7.80–10.67)	11.87 ± 0.66 (10.45–13.28)
Group effect	*F*(1, 14) = 0.483, *P* = 0.499, ES = 0.033	*F*(1, 14) = 0.228, *P* = 0.641, ES = 0.016	*F*(1, 14) = 18.692, ***P***** = 0.001**, ES = 0.572
Time effect	mCIMT: *F*(2, 30) = 128.614, ***P***** < 0.001**, ES = 0.896 control: *F*(2, 28) = 75.246, ***P***** < 0.001**, ES = 0.843		
Time*group	*F*(2, 28) = 3.650, ***P***** = 0.039**, ES = 0.207		
**GRES**			
mCIMT	1.13 ± 0.17 (0.78–1.49)	2.80 ± 0.22 (2.32–3.28)	4.13 ± 0.29 (3.51–4.76)
Control	1.27 ± 0.15 (0.94–1.60)	2.53 ± 0.24 (2.03–3.04)	3.40 ± 0.24 (2.90–3.90)
Group effect	*F*(1, 14) = 0.272, *P* = 0.610, ES = 0.019	*F*(1, 14) = 0.713, *P* = 0.413, ES = 0.048	*F*(1, 14) = 4.193, *P* = 0.060, ES = 0.230
Time effect	mCIMT: *F*(2, 30) = 110.085, ***P***** < 0.001**, ES = 0.880 control: *F*(1.458, 20.406) = 51.070, ***P***** < 0.001**, ES = 0.785		
Time*group	*F*(2, 28) = 3.535, ***P***** = 0.043**, ES = 0.202		

*AMS, Active Movement Scale; MSS, Mallet Shoulder Scale; GRES, Gilbert-Raimondi Elbow Scale. The bold P value < 0.05, considered statistically significant.*

**FIGURE 3:**
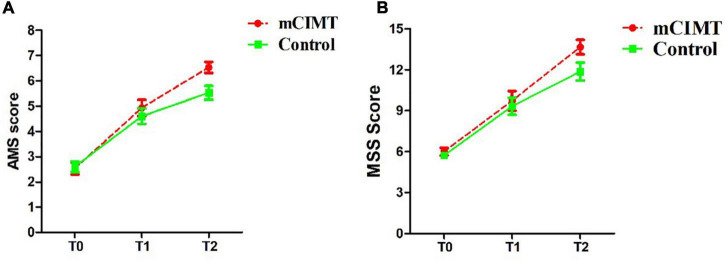
The score (mean ± SE) of AMS **(A)** and MSS **(B)** in the two groups over time. The mCIMT group improved more than the control group. Differences were significant at 6 months follow-up. AMS, Active Movement Scale; MSS, Mallet Shoulder Scale; T0, Acquisition phase; T1, 3 months post-treatment; T2, 6 months post-treatment.

For the internal factor time, the analysis of the AMS revealed a significant effect in both groups [mCIMT: *F*(2, 30) = 128.614, *P* < 0.001, ES = 0.896; control: *F*(2, 28) = 75.246, *P* < 0.001, ES = 0.843]. Similarly, the analysis of the MSS [mCIMT: *F*(2, 30) = 128.614, *P* < 0.001, ES = 0.896; control: *F*(2, 28) = 75.246, *P* < 0.001, ES = 0.843] and GRES [mCIMT: *F*(2, 30) = 110.085, *P* < 0.001, ES = 0.880; control: *F*(1.458, 20.406) = 51.070, *P* < 0.001, ES = 0.785] both revealed a significant effect. These data suggest that children in both groups exhibited significant improvements in upper limb muscle strength, shoulder function and elbow function over time (Figure 3).

No significant, differences were found in baseline (acquisition phase) AMS, MSS, or GRES between the control and mCIMT groups [*F*(1, 14) = 0.062, *P* = 0.086; *F*(1, 14) = 0.483, *P* = 0.499; *F*(1, 14) = 0.272, *P* = 0.610]. No significant differences in AMS, MSS, or GRES scores were observed between groups at the 3-month follow-up time point [*F*(1, 14) = 0.565, *P* = 0.465; *F*(1, 14) = 0.228, *P* = 0.641; *F*(1, 14) = 0.713, *P* = 0.413; *Z* = −0.666, *P* = 0.505]. However, at the 6-month follow-up time point, the mCIMT group exhibited significantly greater improvements in AMS (6.53 ± 0.22 vs. 5.53 ± 0.27) and MSS (13.67 ± 0.53 vs. 13.67 ± 0.53) scores relative to the control group [AMS: *F*(1, 14) = 8.077, *P* = 0.013, ES = 0.366; MSS: *F*(1, 14) = 18.692, *P* = 0.001, ES = 0.572]. These data suggest that mCIMT is superior to traditional rehabilitative treatment as a means of enhancing long-term upper limb functionality (Table 2).

The data of RHS values for patients in the mCIMT and control groups at each time point (baseline, 3-month follow-up, 6-month follow-up) are summarized in Table 3, which were ranked ordinal data. Rank sum test was used for the RHS data. In baseline (acquisition phase), 3-month follow-up time point and 6-month follow-up time point, no significant differences were found between the mCIMT and control groups (*Z* = −0.336, −0.666, −1.874; *P* > 0.05).

**TABLE 3:** RHS measure at each time-point between the two groups, and statistical comparison (rank sum test).

	RHS	Baseline						3-month follow-up						6-month follow-up					
				
	*N*	0	1	2	3	4	5	0	1	2	3	4	5	0	1	2	3	4	5
mCIMT	16	0	6	8	2	0	0	0	0	3	8	4	1	0	0	0	2	8	6
Control	15	1	7	7	0	0	0	0	0	5	7	3	0	0	0	1	3	10	1
Z		−0.336						−0.666						−1.874					
P		0.737						0.505						0.061					

*RHS, Raimondi Hand Scale.*

## Discussion

This RCT have explored the impact of mCIMT on children suffering from residual upper limb dysfunction as a consequence of uBPI. Objective analyses of the utility of the mCIMT approach were conducted using the AMS, MSS, GRES, and RHS scales, ultimately revealing that both mCIMT and conventional rehabilitation can effectively improve muscle strength and shoulder, elbow, and hand function in uBPI patients, with mCIMT being superior to conventional rehabilitation.

mCIMT is a rehabilitation strategy that was developed in the 1980s through behavioral studies of monkeys (Ostendorf and Wolf, 1981; Acan et al., 2018), focusing on observed learned disuse and corresponding shaping techniques. Cutting off the afferent nerve of one forelimb in a monkey has been shown to result in the ineffective use of that limb, with the monkeys then adapting to the use of other limbs to navigate their environment through a process known as learned disuse. Shaping technologies refer to the use of functional training efforts that enable the affected limb to conduct concentrated, repetitive activities of daily living (Williams and Wiskonsin, 1980). Learned disuse can be overcome within a few days through the restriction of the movement of the monkey’s healthy limbs, thus forcing them to utilize the affected limbs. The goal of mCIMT is to overcome such learned disuse while improving the functionality of affected limbs through a forced shift in motivation outcomes (Fritz et al., 2012). Previously, mCIMT has been employed successfully to overcome upper limb impairment following stroke, and it has become the most widely studied interventional approach for stroke patient treatment (Rocha et al., 2021). Moreover, mCIMT has been employed to treat children with hemiplegia, improving upper limb sensory and motor function, with a growing body of evidence supporting the efficacy of mCIMT treatment (Anita et al., 2013; Islam et al., 2014).

Brachial plexus injuries are similar to the mCIMT deafferentation model, with both involving peripheral nerve injuries resulting in impaired motor function (Pondaag and Malessy, 2014). For children suffering from such injuries, mCIMT was applied in a manner similar to that used previously in experimental macaques. During the early stages, brachial plexus injuries result in learned upper limb non-use. By restricting the healthy limb, the acquired disuse of the affected upper limb can thus be overcome through plastic techniques. Overcoming learned non-use in patients brachial plexus nerve injury patients can thus be achieved in a manner similar to that in the context of nerve deafferentation, and we thus posit that mCIMT is suitable for brachial plexus injury treatment.

Both conventional rehabilitation and mCIMT were associated with similar levels of improvement at the 3-month follow-up time point in this study, whereas at the 6-month time point, patients in the mCIMT group exhibited significantly better outcomes. This suggests that mCIMT treatment is associated with distinct long-term improvements in residual upper limb dysfunction in children with uBPI. These results may suggest that the benefits associated with such treatment are further improved by the additional mastery of the learned skills. At begin, restricting healthy upper limb movement in children with unilateral cerebral palsy may lead to provisional movement regulation disorder and reduced active movement (Yvonne et al., 2013). But as time goes on, children show the ability to adjust such restriction and the advantages of mCIMT become more apparent. That is, why we observed significantly greater improvements in AMS and MSS scores in the mCIMT group relative to the control group at 6-month time point. In summary, mCIMT is associated with long-term benefits to the rehabilitation of residual upper limb dysfunction associated with uBPI in infants.

There are certain limitations to this analysis. For one, restriction methods were determined on an individual basis. Moreover, to prevent adverse effects on healthy side movement as a consequence of restriction for 4 h, the unaffected limb underwent hand-arm intensive bimanual therapy for 30 min. The number of cases included in this study was relatively small, and these results may thus not fully reflect the actual clinical efficacy of mCIMT treatment, with the differences in therapeutic outcomes potentially being attributable to the age of the treated child. As such, additional large-scale studies will be essential to confirm whether mCIMT is beneficial as a treatment for upper limb dysfunction in children diagnosed with brachial plexus injury.

## Data Availability Statement

The raw data supporting the conclusions of this article will be made available by the authors, without undue reservation.

## Ethics Statement

The studies involving human participants were reviewed and approved by the Ethics Committee of the First Affiliated Hospital of Anhui Medical University. Written informed consent was obtained from the individual(s), and minor(s)’ legal guardian/next of kin, for the publication of any potentially identifiable images or data included in this article.

## Author Contributions

DW contributed to the conception of the study. ZC performed the data analyses and wrote the manuscript. LL contributed significantly to analysis and manuscript preparation. XC and SZ provided the system rehabilitation training for the patients. HZ helped perform the analysis with constructive discussions. All authors contributed to the article and approved the submitted version.

## Conflict of Interest

The authors declare that the research was conducted in the absence of any commercial or financial relationships that could be construed as a potential conflict of interest.

## Publisher’s Note

All claims expressed in this article are solely those of the authors and do not necessarily represent those of their affiliated organizations, or those of the publisher, the editors and the reviewers. Any product that may be evaluated in this article, or claim that may be made by its manufacturer, is not guaranteed or endorsed by the publisher.

## Abbreviations

mCIMT: modified Constraint-Induced Movement Therapy
uBPI: brachial plexus injury
AMS: Active Movement Scale
MSS: Mallet Shoulder Scale
GRES: Gilbert-Raimondi Elbow Scale
RHS: Raimondi Hand Scale.

## References

[B1] Acan A. E. Gursan O. Demirkiran N. D. Havitcioglu H. (2018). Late treatment of obstetrical brachial plexus palsy by humeral rotational osteotomy and lengthening with an intramedullary longation nail. *Acta Orthop. Traumatol. Turc.* 52 75–80. 10.1016/j.aott.2017.03.019 28495173 PMC6136309

[B2] Al-Qattan M. M. (2003). Assessment of the motor power in older children with obstetric brachial plexus palsy. *J. Hand Surg. Br.* 28 46–49. 10.1054/jhsb.2002.0831 12531668

[B3] Anita C. Sheffali G. Madhulika K. Moja L. Gatti R. (2013). Efficacy of modified constraint induced movement therapy in improving upper limb function in children with hemiplegic cerebral palsy: a randomized controlled trial. *Brain Dev.* 35 870–876. 10.1016/j.braindev.2012.11.001 23238223

[B4] Blaauw G. Kortleve J. Muhlig R. (1998). *Evaluation and Management of Obstetrical Brachial Plexus Lesions.* Amsterdam: Workshop on Obstetrical Plexus Lesions.

[B5] Brucker J. Laurent J. P. Lee R. Shenaq S. Parke J. Solis I. Cheek W. (1991). Brachial plexus birth injury. *J. Neurosci. Nurs.* 23 374–380. 1839546 10.1097/01376517-199112000-00006

[B6] Curtis C. Stephens D. Clarke HM. Andrews D. (2002). The active movement scale: an evaluative tool for infants with obstetrical brachial plexus Palsy. *J. Hand Surg. Am.* 27 470–478. 10.1053/jhsu.2002.32965 12015722

[B7] Fritz S. L. Butts R. J. Wolf S. L. (2012). Constraint-induced movement therapy:from history to plasticity. *Expert Rev.* 12 191–198. 10.1586/ern.11.201 22288674

[B8] Gert K. Janne M. V. Erwin E. H. (2015). Constraint-induced movement therapy: from history to plasticity. *Lancet Neurol.* 14 224–234. 25772900

[B9] Goubier J. N. Maillot C. Asmar G. Frederic T. (2019). Partial ulnar nerve transfer to the branch of the long head of the triceps to recover elbow extension in C5, C6 and C7 brachial plexus palsy. *Injury* 50 S68–S70. 10.1016/j.injury.2019.10.052 31690498

[B10] Heise C. O. Martins R. Siqueira M. (2015). Neonatal brachial plexus palsy: a permanent challenge. *Arq. Neuropsiquiatr.* 73 803–808. 10.1590/0004-282X20150105 26352501

[B11] Hoeksma A. F. Steeg A. M. Nelissen R. G. Ouwerkerk W. J. Lankhorst G. J. Jong B. A. (2004). Neurological recovery in obstetric brachial plexus injuries: an historical cohort study. *Dev. Med. Child Neurol.* 46 76–83. 10.1017/s0012162204000179 14974631

[B12] Islam M. Nordstrand L. Holmström L. Kits A. Forssberg H. Eliasson A. C. (2014). Is outcome of constraint-induced movement therapy in unilateral cerebral palsy dependent on corticomotor projection pattern and brain lesion characteristics? *Dev. Med. Child Neurol.* 56 252–258. 10.1111/dmcn.12353 24341408

[B13] Joshua M. Scott H. (2014). Evaluation and management of brachial plexus birth palsy. *Orthop. Clin. North Am.* 45 225–232. 24684916 10.1016/j.ocl.2013.12.004

[B14] Knapp H. Taub E. Berman A. (1963). Movements in monkeys with deafferented forearms. *Exp. Neurol.* 7 305–315. 14033714 10.1016/0014-4886(63)90077-3

[B15] Luo T. D. Levy M. L. Li Z. (2021). *Brachial Plexus Injuries. StatPearls.* Treasure Island (FL): StatPearls Publishing. 29493930

[B16] Ostendorf C. G. Wolf S. L. (1981). Effect of forced use of the upper extremity of a hemiplegic patient on changes in function. a single-case design. *Phys. Ther.* 61 1022–1028. 10.1093/ptj/61.7.1022 7243897

[B17] Polcaro L. Charlick M. Daly D. T. (2019). *Anatomy, Head and Neck, Brachial Plexus. Stat Pearls.* Treasure Island (FL): StatPearls Publishing. 30285368

[B18] Pondaag W. Malessy M. J. A. (2014). The evidence for nerve repair in obstetric brachial plexus palsy revisited. *Biomed Res. Int.* 2014:434619. 10.1155/2014/434619 24551845 PMC3914347

[B19] Praveen B. Navin B. (2009). Motor grading of elbow flexion – is Medical Research Council grading good enough? *J. Brachial. Plex. Peripher. Nerve Inj.* 4:3. 10.1186/1749-7221-4-3 19439090 PMC2694805

[B20] Prigent N. Q. Romana C. (2013). Multidisciplinary management in children with obstetric brachial plexus injury (OBPI). *Pédiatrie* 56 e288–e294.

[B21] Rocha L. S. O. Gama G. C. B. Rocha R. S. B. Rocha L. B. Dias C. P. Santos L. L. S. (2021). Constraint induced movement therapy increases functionality and quality of life after stroke. *J. Stroke Cerebrovasc. Dis.* 30:105774. 10.1016/j.jstrokecerebrovasdis.2021.105774 33848906

[B22] Sibbel S. E. Bauer A. E. James M. A. (2014). Late reconstruction of brachial plexus birthpalsy. *J. Pediatr. Orthop.* 34 S57–S62. 25207738 10.1097/BPO.0000000000000290

[B23] Smith B. W. Daunter A. K. Yang L. J. S. Wilson T. (2018). An update on the management of neonatal brachial plexus palsy—replacing old paradigms. *JAMA Pediatr.* 172 585–591. 10.1001/jamapediatrics.2018.0124 29710183

[B24] Smith K. Patel V. (2016). Congenital brachial plexus palsy. *Paediatr. Child Health* 26 152–156.

[B25] Williams A. Wiskonsin S. (1980). Somatosensory deafferentation research with monkeys: implications for rehabilitation medicine Behavioral Psychology in Rehabilitation medicine. *Clin. Appl.* 2 371–401.

[B26] Yvonne G. Pauline A. Alexander C. (2013). Motor learning curve and long-term effectiveness of modified constraint-induced movement therapy in children with unilateral cerebral palsy: a randomized controlled trial. *Res. Dev. Disabil.* 34 923–931. 10.1016/j.ridd.2012.11.011 23291509

[B27] Zielinski I. M. Delft R. Voorman J. M. Geurts A. C. H. Steenbergen B. Aarts P. B. M. (2021). The effects of modified constraint-induced movement therapy combined with intensive bimanual training in children with brachial plexus birth injury: a retrospective data base study. *Disabil. Rehabil.* 43 2275–2284. 10.1080/09638288.2019.1697381 31814455

